# Structural and Superconducting Properties of Thermal Treatment-Synthesised Bulk YBa_2_Cu_3_O_7−δ_ Superconductor: Effect of Addition of SnO_2_ Nanoparticles

**DOI:** 10.3390/ma12010092

**Published:** 2018-12-28

**Authors:** Nur Nabilah Mohd Yusuf, Mohd Mustafa Awang Kechik, Hussein Baqiah, Chen Soo Kien, Lim Kean Pah, Abdul Halim Shaari, Wan Nur Wathiq Wan Jusoh, Safia Izzati Abd Sukor, Mustafa Mousa Dihom, Zainal Abidin Talib, Roslan Abd-Shukor

**Affiliations:** 1 Department of Physics, Faculty of Science, Universiti Putra Malaysia, UPM Serdang 43400, Malaysia; nabilaupm@gmail.com (N.N.M.Y.); husseinbaqiah@gmail.com (H.B.); chensk@upm.edu.my (C.S.K.); limkp@upm.edu.my (L.K.P.); ahalim@upm.edu.my (A.H.S.); athiq05@gmail.com (W.N.W.W.J.); safiasukor@gmail.com (S.I.A.S.); MD@yahoo.com (M.M.D.); zainalat@upm.edu.my (Z.A.T.); 2 School of Applied Physics, Faculty of Science and Technology, Universiti Kebangsaan Malaysia, Bangi 43600, Malaysia; ras@ukm.edu.my

**Keywords:** additions, YBa_2_Cu_3_O_7−δ_, SnO_2_, bulk superconductor, thermal treatment method

## Abstract

YBa_2_Cu_3_O_7−δ_ (Y-123) bulk superconductors with the addition of (0.0, 0.2, 0.4, 0.6, 0.8, and 1.0 wt.%) SnO_2_ nanoparticles were synthesised via a thermal treatment method. The influence of SnO_2_ addition on the superconducting properties by means of critical temperature, *T*_c_, AC susceptibility, phase formation and microstructures, including its elemental composition analysis, were studied. Sharp superconducting transition, ∆*T*_c_, and diamagnetic transition were obtained for all SnO_2_-added samples. It was observed that sample *x* = 0.4 with a Y-123 phase percentage of 95.8% gives the highest *T*_c_, smallest ∆*T*_c_, and the sharpest diamagnetic transition in the normalised susceptibility curves. The microstructure also showed an excess of Sn precipitates on the sample’s surface at *x* = 0.8 and above. As such, the best superconducting properties were observed at *x* = 0.4 SnO_2_ addition inside the Y-123 host sample.

## 1. Introduction

The YBa_2_Cu_3_O_7−δ_ (Y-123) superconductor has been widely studied since Paul Chu et al. first discovered it in 1986 [1]. This compound belongs to the type II superconductors, where the superconducting state is limited by three main factors: critical temperature, *T_c_*, critical magnetic field, *H_c_*_2_, and critical current, *J_c_*. The enhancement of these parameters is the key for investing this material in technology applications [2]. Unfortunately, bulk Y-123 suffers from low grain boundary conductivity, i.e., weak links and poor flux pinning, resulting in low *J_c_* in the presence of magnetic field [3], and its *T*_c_ is sensitive to oxygen content in the system [4]. Therefore, many efforts have been devoted to overcoming these issues in order to achieve the *J*_c_ and *T*_c_ required for technological applications [5,6].

A variety of research has been done focussing on improving the superconducting properties of Y-123 ceramic by altering the techniques with which it can be synthesised and adding impurities that act as artificial pinning centres in the sample. A previous study by Dihom et al. gave a remarkable result of *T*_c-onset_ = 93 K for pure Y-123 prepared using a thermal treatment method in which only metal nitrates, polyvinyl pyrrolidone (PVP) and deionised water were used [7]. The employment of this thermal treatment method in the field of superconductors is still new, but has a lot of potential due to its simple procedure, which makes use of a capping agent during the synthesis process [7,8,9,10].

Conducting and semiconducting nanomaterial impurities introduced into the Y-123 system were found to enhance its transport properties. These impurities are expected to act as pinning centres and/or reduce the impact of the weak link of the grain boundaries [11,12,13,14,15]. For instance, conductive materials of carbon nanotubes (CNTs) added to Y-123 synthesized using a co-precipitation process acted as pinning centres and resulted in an increase of *J*_c_ from 11 A/cm^2^ for *x* = 0.0 to 477 A/cm^2^ for the sample with *x* = 0.2 wt.% [14]. TiO_2_ semiconducting nanoparticles were added to Y-123 prepared by solid-state reaction. The TiO_2_ nanoparticles were unreacted with the Y-123 matrix and were observed in XRD patterns. This resulted in increasing self-field *J*_c_ due to the enhancement flux pinning ability [16]. On the other hand, addition of HfO_2_ nanoparticles to Y-123 fabricated via a modified combustion method formed YBa_2_HfO_5.5_ phase, which also acts as a pinning centre for Y-123, resulting in the enhancement of *J_c_* [5]. Sn and SnO_2_ are frequently introduced into Y-123 thin films fabricated by metal organic deposition process. These materials form Sn-based nanomaterials in the Y-123 matrix that can act as effective pinning centres [17,18,19]. Uniform distributed Sn-based reacted phase was also observed when SnO_2_ was added into melt texture grown bulk Y-123 [20]. Following up the same motivation as previous research, we report the investigation into the microstructural and superconducting properties of Y-123 synthesized by the thermal treatment method and with the addition of *x* = 0.0, 0.4, 0.8 and 1.0 wt.% SnO_2_ nanoparticles.

## 2. Materials and Methods 

YBa_2_Cu_3_O_7−δ_ (Y-123) powders were prepared by using a thermal treatment method similar to that reported by Dihom et al. [7]. Stoichiometric weights of metal nitrates Y(NO_3_)_3_·6H_2_O (Alfa Aesar, Haverhill, MA, USA, 99.90%), Ba(NO_3_)_2_ (Alfa Aesar, 99.95%), and Cu(NO_3_)_2_·2.5H_2_O (Alfa Aesar, 98%) were dissolved in 300 mL of 2% polyvinyl pyrrolidone (Sigma Aldrich, Steinheim, Germany, PVP) aqueous solution and stirred together at 80 °C for 2 h The solution was dried in the oven for 24 h at 110 °C. The remaining solid-like green gel was ground into fine powder and calcined at 600 °C for 4 h and 910 °C for 24 h with intermediate grinding. The obtained Y-123 powder was reground and mixed with *x* = 0.0, 0.4, 0.8 and 1.0 wt.% SnO_2_ nanoparticles (Nanostructured & Amorphous Materials, Inc., Houston, TX, USA, average particle size 55 nm). The mixed powders were pressed into pellets and sintered at 980 °C for 24 h under the flow of oxygen.

The structure and phase formation were studied using Phillip X-ray diffractometer (XRD) (PANalytical, Lelyweg, Almelo, The Netherlands. with Cu Kα radiation source and analysed via Rietveld refinement embedded in the X’pert Plus HighScore software (PANalytical, Lelyweg, Almelo, The Netherlands). Scanning electron microscopy (SEM) and energy dispersive X-ray (EDX) were used to study the morphologies and elemental distribution of the sample surface. The Y-123 pellet was carefully broken using a pestle and mortar into small pieces. The SEM and EDX were performed on both surfaces and the cross-section of the small piece of the pellet. A standard four-point probe method was used to measure the electrical resistance of the bulk sample, while the measurement of AC susceptibility was performed on a rectangular bar-shaped sample using a CryoBind (Cryogenic Balanced Inductive Detector, CryoBIND, Zagreb, Croatia) SR830 lock-in amplifier at an applied field of 1 Oe. 

## 3. Results and Discussion

### 3.1. X-ray Diffraction Analysis

**Figure 1:**
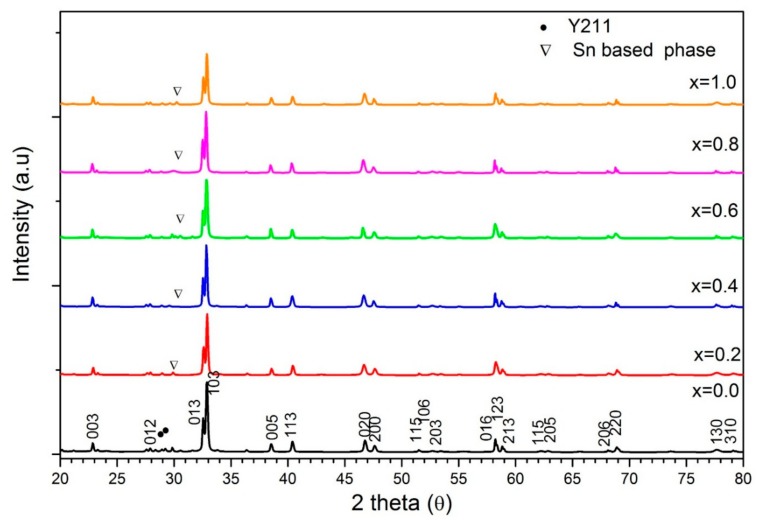
XRD diffractograms of Y-123 with *x* = 0.0, 0.2, 0.4, 0.6, 0.8, and 1.0 wt.% SnO_2_ addition.

XRD patterns of Y-123 with SnO_2_ addition are shown in Figure 1. The XRD analysis of the pure sample matches Y-123 reference ICSD NO. 98-003-6464, with a superconducting orthorhombic phase and space group symmetry Pmmm. Most of the (hkl) peaks obtained are in good agreement with Y-123, especially the highest peak (103) at 32.8° [15,21]. Extra peaks are also observed between 27° and 31°, corresponding to the Y-211 secondary phase in the pure sample. It is noted that there are no considerable changes in the polycrystalline patterns of the diffractograms upon the addition of SnO_2_, except for extra small peaks found at 30.16° and 43.3°. These peaks were controversially indexed. While some groups assigned these peaks to BaSnO_2_ [19,22], Choi et al. indicate that these peaks belong to Y-Ba-Sn-O [18]. However, it can be concluded that Sn was not incorporated into the Y-123 crystal, and instead participated in the form of Sn-based secondary phases. This is a good indication that there was no substitution of Sn in the Y-123 system [15].

Rietveld refinement showed that the addition of SnO_2_ increases the percentage of Y-123 phase in the sample, hence reducing the percentage of Y-211 phase. Meanwhile, the insignificant changes in the lattice parameters *a*, *b* and *c* further support that there was no substitution of Sn in the sample, which is in good agreement with the results reported in [23]. Orthorhombicity of the Y-123 phase was calculated using the lattice parameter differences (a−b)/(a+b) and is tabulated in Table 1 [15]. The average crystallite size shown in table was estimated from peaks located at 2θ = 22.8°, 32.8°, 38.5°, 40.4° and 46.7° using the Scherrer equation in [24].

(1) $$p=\frac{k\lambda }{b\cos\theta }$$

where *p* is the ‘true’ crystallite size, *k* is the Scherrer constant (~0.9), *λ* is the X-ray radiation wavelength (*λ*_Cu_ = 0.15418 nm), *b* = *b*_obs_ − *b*_std_, where *b*_obs_ is the full width at half maximum and *b*_std_ is related to the broadening of the XRD instrument. The crystallite size of samples was unsystematically changed upon addition of SnO_2_. 

### 3.2. Scanning Electron Microscopy and Elemental Analysis 

**Figure 2:**
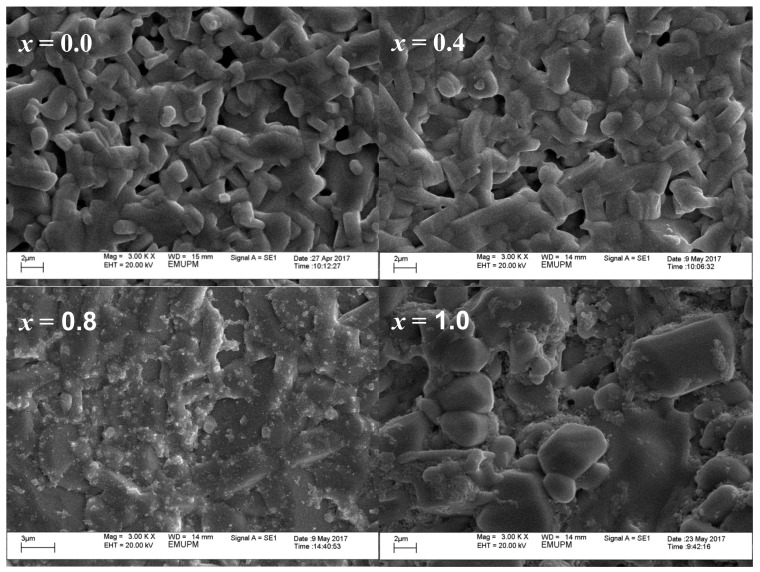
SEM images of Y-123 sample surface at *x* = 0.0, 0.4, 0.8 and 1.0 wt.% SnO_2_ addition.

**Figure 3:**
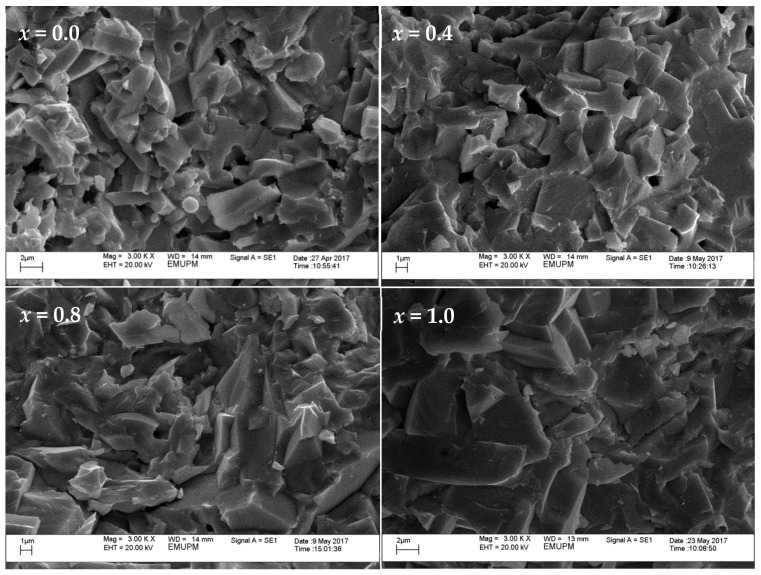
SEM image of Y-123 cross-section at *x* = 0.0, 0.4, 0.8 and 1.0 SnO_2_ addition.

**Figure 4:**
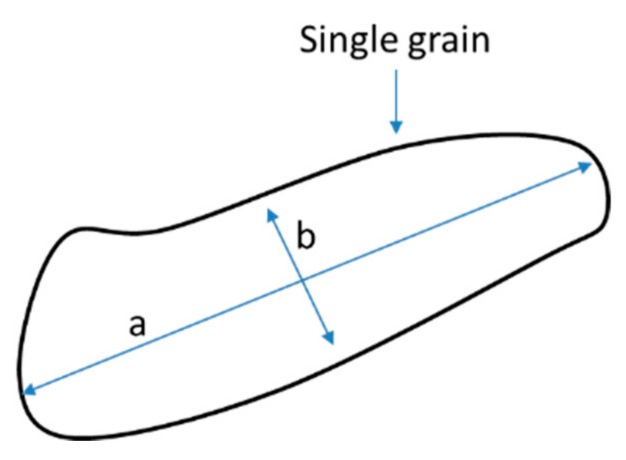
Schematic diagram for the dimensions in which the average grain size is measured.

**Figure 5:**
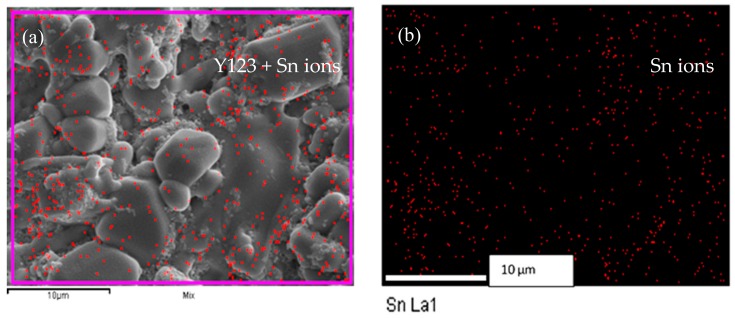
(**a**) SEM image of the surface of Y-123 at *x* = 1.0 together with EDX mapping Sn ions (red dots) and (**b**) EDX mapping of Sn ions without Y-123 matrix.

The SEM images in Figure 2 and Figure 3 reveal the morphologies of both the surface and cross-section of the bulk samples, respectively, at (3000×) magnification. Figure 2 shows a granular grain growth for pure Y-123 ceramics with an average grain size of 1.6 μm. It is apparent that both Figure 2 and Figure 3 show an enhancement of grain size and compactness structures as the SnO_2_ addition increases. The grains also seem to elongate more at one side, forming a rectangular-like structure. The average grain size was measured using 133 to 152 selected grains by considering both dimension of the grains, as illustrated in Figure 4, and the results obtained were 1.6 μm, 1.8 μm, 4.6 μm and 4.9 μm for *x* = 0.0, 0.4, 0.8 and 1.0, respectively. From the EDX elemental mapping, Sn ion precipitates started to appear at *x* = 0.8 and tended to agglomerate at the grain boundaries on the surface of the sample. The elemental compositional ratios for all samples are also relatively close to the 1:2:3 ratio of YBa_2_Cu_3_O_7−δ_. The EDX mapping of Sn ions on the surface and cross-section of *x* = 1.0 is illustrated in Figure 5. The Sn ion mapping, represented by red dots on the surface of the *x* = 1.0 sample, further confirms the agglomeration of Sn-based phases at the grain boundaries, while the Sn ion mapping on the cross-section reveals the presence of randomly scattered Sn across the cross-section.

### 3.3. Electrical Resistance Measurement

**Figure 6:**
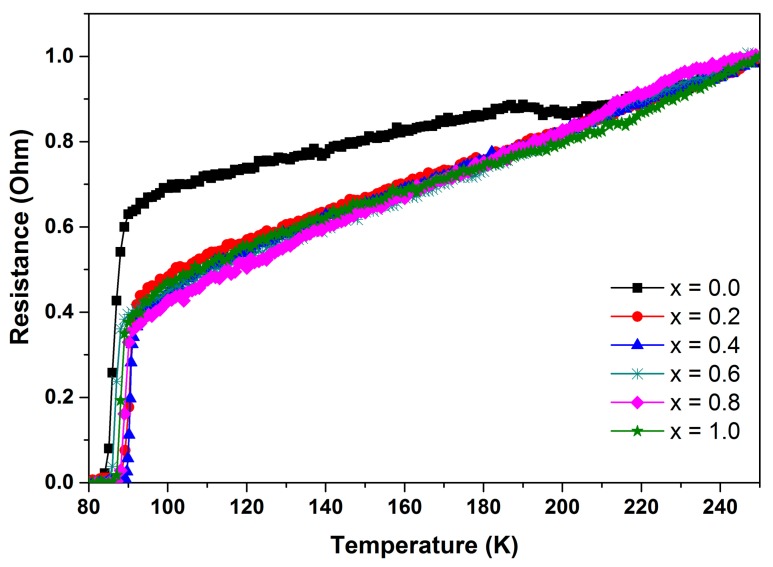
Resistance versus temperature graph for Y-123 with *x* = 0.0, 0.2, 0.4, 0.6, 0.8, and 1.0 SnO_2_ addition.

Figure 6 shows the electrical resistance measurement on all samples exhibiting metallic behaviour in the normal state. The first drop of resistance in the superconducting transition was labelled as *T*_c-onset_, and the temperature at which resistance starts to zero is the *T*_c-offset_. The critical temperature *T_c-onset_* was attained at 90, 92, 91, 89, 90 and 90 K, while the *T*_c-offset_ was 83, 88, 88, 85, 86 and 86 K for samples *x* = 0.0, 0.2, 0.4, 0.6, 0.8 and 1.0, respectively. From Table 2, it is figured that there is not much change in the *T*_c-onset_ and *T*_c-offset_ of the sample. However, superconducting transition width, (*∆T*_c_ = *T*_c-onset_ − *T*_c-offset_), listed in Table 2 decreased upon the addition of SnO_2_, implying that the grains’ connectivity had been improved [25]. This may be attributed from a more homogeneous oxygenation of grains in the sample achieved by the addition of SnO_2_ in Y-123 sample [26]. SnO_2_ nanoparticles were previously added at one concentration (*x* = 0.2 wt.%) into Y-123 synthesized using the solid-state reaction method [23]. By comparing our results with those obtained by Salama et al., it can be noted that the *T*_c-onset_, *T*_c-offset_ and *∆T* in of the pure sample in their work are better than those in the current work. However, the superconducting properties of the SnO_2_ added to the thermal treatment-synthesized Y-123 at *x* = 0.2, i.e., this work, were improved, and were even better than those reported in [23], see Table 2. 

### 3.4. Alternating Current Susceptibility

**Figure 7:**
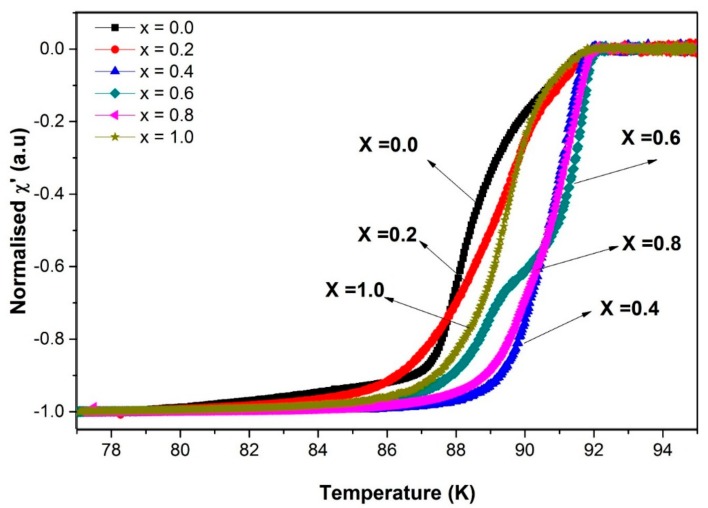
The real part of the normalised susceptibility curve for Y-123 with *x* = 0.0, 0.2, 0.4, 0.6 0.8, and 1.0 wt.% SnO_2_ addition.

**Figure 8:**
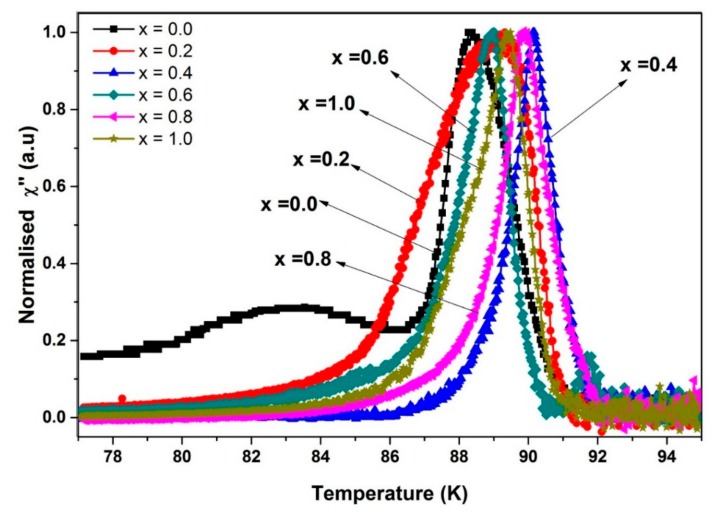
The imaginary part of the normalised susceptibility curve for Y-123 with *x* = 0.0, 0.2, 0.4, 0.6, 0.8, and 1.0 wt.% SnO_2_ addition.

The normalised AC susceptibility for both real χ′ and imaginary χ″ parts are presented in Figure 7 and Figure 8, respectively. The information regarding the diamagnetic transition of bulk superconductor can be obtained from the real part χ′ [27]. Double step transition in this part is due to the weak link behaviour of the superconducting sample [28]. It is noted that the first drop at higher temperature, *T*_c-onset_, is related to intragranular shielding, while the second drop at a lower temperature, *T*_cj_, is related to intergranular shielding [29]. In Figure 7, sample *x* = 0.0 exhibited the double step behaviour, which was improved with the addition of SnO_2_. At *x* = 0.4, the transition became steeper, indicating reduced weak links and a stronger interconnectivity between the grains. However, the transition starts to broaden at *x* = 0.8 and *x* = 1.0, suggesting that *x* = 0.4 is the optimum weight percentage for SnO_2_ addition. This broadened curve is caused by the magnetic flux penetrating the sample surface when there is a decrease in the screening current required to exclude the flux [30]. From *T*_c-onset_ and the phase-lock in the temperature, *T*_cj_, tabulated in Table 3, maximum Josephson current, *I*_0_, can be estimated using the Ambegaokar-Baratoff theory in the following equation [31],

(2) $$I_{0}=\left(1.57\;\times 10^{-8}\;A/K\right)\;\;\frac{T_{\mathrm{c-onset}}{}^{2}}{T_{\mathrm{c-onset}}\;-\;\;T_{\mathrm{cj}}}$$

From the estimated value of *I*_0_, *x* = 0.4 gives the highest value with 664 μA compared to *x* = 0.0, 146 μA. This held true for the imaginary part, where the coupling effect of the grains can be observed based on intergranular peaks. Figure 8 shows that the intercoupling peak *T*_p_ shifted towards a higher temperature when *x* = 0.4, and slowly shifted towards a lower temperature when addition increases. The peak shifting towards a higher temperature demonstrates a stronger pinning and stronger intergranular critical current density, suggesting that *x* = 0.4 is the best weight percentage for SnO_2_ in Y-123 among the additions. It is noted that the pure sample has another broad peak at ~83 K, believed to be due to a secondary phase in which second superconducting transition occurs [32]. Previous literature has reported that the second superconducting transition may originate from the variation of the order parameter of the superconducting state, which is related to the internal degrees of freedom of the Cooper pairs [33].

## 4. Conclusions

Bulk Y-123 superconductors have been successfully synthesised via a thermal treatment method with SnO_2_ addition at *x* = 0.0, 0.2, 0.4, 0.6, 0.8, and 1.0 wt.%. It is well observed that the samples showed metallic behaviour during the normal state and exhibit sharper superconducting transition with the presence of SnO_2_ in the resistance-temperature graph. Critical current temperature, *T*_c-onset_, and phase-lock in temperature, *T*_cj_, obtained from the AC susceptibility curves also showed a slight enhancement up to *x* = 0.4, which slowly decreased at higher addition. Maximum Josephson current, *I*_o_, was also calculated and resulted in the highest value of 664 μA at *x* = 0.4. From XRD analysis, the major phase of Y-123 was found in all samples, while the secondary phase of Y211 was reduced at *x* = 0.4 and disappeared at *x* = 0.8 and 1.0. Sn precipitates were also observed in the SEM images on the surface of the bulk sample at *x* = 0.8 and 1.0, which was further confirmed by EDX mapping. Hence, the sample with *x* = 0.4 SnO_2_ addition exhibited the best superconducting properties among the samples in this study.

## Figures and Tables

**Table 1:** Phase percentage of Y-123, Y211 and SnO_2_, including the lattice parameters *a*, *b*, and *c*, orthorhombicity and crystallite size of the Y-123 samples at *x* = 0.0, 0.2, 0.4, 0.6, 0.8, and 1.0 wt.% SnO_2_ addition.

SnO_2_ Addition (*x* = wt.%)	Y123%	Y211%	Sn Based Phase%	a/Å	b/Å	c/Å	Orthorhombicity (10^−3^)	Crystallite Size (nm)
0.0	91.5	8.5	-	3.8201(2)	3.8846(3)	11.676(1)	8.372	91 ± 11
0.02	94.2	1.8	4.0	3.8211	3.8842	11.6760	8.189	100 ± 28
0.4	95.8	0.1	4.2	3.8232(1)	3.8862(2)	11.6793(7)	8.185	98 ± 28
0.06	91.5	-	8.5	3.8239	3.8864	11.6840	8.067	170 ± 65
0.8	93.8	-	6.2	3.8218(1)	3.8864(2)	11.6740(8)	8.381	110 ± 26
1.0	94.0	-	6.0	3.8232(1)	3.8863(2)	11.6793(8)	8.185	95 ± 20

**Table 2:** *T*_c-onset_, *T*_c-offset_, and *∆T*_c_ for Y-123 at *x* = 0.0, 0.2, 0.4, 0.6, 0.8, and 1.0 wt.% SnO_2_ addition in this work, and in = 0.0 and 0.2 reported in [23].

SnO_2_ Addition (*x* = wt.%)	*T*_c-onset_ (K)	*T*_c-offset_ (K)	∆*T*_c_ (K)	Ref.
**0.0**	90	83	7	This work
**0.2**	92	88	4	This work
**0.4**	91	88	3	This work
**0.6**	89	85	4	This work
**0.8**	90	86	4	This work
**1.0**	90	86	4	This work
**0.0**	87	92	5	[23]
**0.2**	67	79	12	[23]

**Table 3:** *H*_ac_, *T*_c-onset_, *T*_cj_, *T*_p_, and *I*_0_ for Y-123 at *x* = 0.0, 0.02, 0.4, 0.06, 0.8, and 1.0 SnO_2_ addition.

SnO_2_ Addition (*x* = wt.%)	*H*_ac_ (Oe)	*T*_c-onset_ (K)	*T*_cj_ (K)	*T*_p_ (K)	*I*_0_ (μA)
**0.0**	1	91.5	90.6	88.3	146
**0.2**	1	91.7	90.9	89.3	147
**0.4**	1	92.0	91.8	90.2	664
**0.6**	1	92.0	90.1	88.9	70
**0.8**	1	92.0	90.8	89.9	111
**1.0**	1	91.8	91.2	89.4	189
